# Differences in microglia morphological profiles reflect divergent emotional temperaments: insights from a selective breeding model

**DOI:** 10.1038/s41398-022-01821-4

**Published:** 2022-03-15

**Authors:** Pamela M. Maras, Elaine K. Hebda-Bauer, Megan H. Hagenauer, Kathryn L. Hilde, Peter Blandino, Stanley J. Watson, Huda Akil

**Affiliations:** The Michigan Neuroscience Institute, University of Michigan School of Medicine, Ann Arbor, MI 48109 USA

**Keywords:** Molecular neuroscience, Predictive markers

## Abstract

Microglia play critical roles in healthy brain development and function, as well as the neuropathology underlying a range of brain diseases. Despite evidence for a role of microglia in affective regulation and mood disorders, little is known regarding how variation in microglia status relates to individual differences in emotionality. Using a selective breeding model, we have generated rat lines with unique temperamental phenotypes that reflect broad emotional traits: bred low responder rats (bLRs) are novelty-averse and model a passive coping style, whereas bred high responder rats (bHRs) are highly exploratory and model an active coping style. To identify a functional role of microglia in these phenotypes, we administered minocycline, an antibiotic with potent microglia inhibiting properties and observed shifts in forced swim, sucrose preference, and social interaction behaviors in bLRs. Using detailed anatomical analyses, we compared hippocampal microglia profiles of bHRs and bLRs and found that although the lines had similar numbers of microglia, selective breeding was associated with a shift in the morphological features of these cells. Specifically, microglia from bLRs were characterized by a hyper-ramified morphology, with longer processes and more complicated branching patterns than microglia from bHRs. This morphology is thought to reflect an early stage of microglia activation and suggests that bLR microglia are in a reactive state even when animals are not overtly challenged. Taken together, our results provide novel evidence linking variation in inborn temperament with differences in the baseline status of microglia and implicate a role for microglia in shaping enduring emotional characteristics.

## Introduction

The expression of emotional behaviors reflects complex interactions between genetic and environmental factors. Within the range of inborn factors, broad personality traits represent a key predictor for emotional resilience or vulnerability [1–3]. Often referred to as temperament, considerable variation exists in the way individuals interact with their environment and respond to challenges [4, 5]. Emotional temperaments represent traits that are highly stable throughout the lifespan and across different conditions and are a risk factor for developing certain psychopathologies [2, 4]. Indeed, individuals with a timid or novelty-averse temperament are at significantly higher risk for developing clinical anxiety and depression [5–8]. Despite the recognition of temperament as a critical individual variable, the neurobiological mechanisms that underlie emotionality traits remain unclear.

Selective breeding models provide a useful tool to study the neurobiology of temperament and its relationship to emotional output. By selectively breeding rats based on locomotor response to novelty, our laboratory has generated lines with opposing temperaments: bred high responders (bHRs) and bred low responders (bLRs) [9]. bHRs are highly exploratory with an active coping style that models an “externalizing temperament”. By contrast, bLRs are novelty-averse and represent a passive or inhibited coping style that models an “internalizing temperament” [3, 9]. These phenotypes are highly heritable [10] and predict a wide range of emotional behaviors [11–16], as well as responsivity to pharmacological and environmental interventions [15, 17–22]. In multiple laboratory assays, bLRs display increased anxiety- and depressive-like behaviors and reduced social exploration and proactive coping [3]. Several lines of evidence from our lab implicate the hippocampus as a critical brain region underlying bHR/bLR phenotypes [14, 20, 23, 24], and a recent meta-analysis of transcriptional studies spanning multiple generations identified broadly divergent gene expression patterns within hippocampus [25]. Interestingly, microglia-related pathways emerged as one of the main functional candidates from this analysis, with bLR hippocampus exhibiting elevated expression of several key genes related to microglial activation and signaling [25].

These findings complement a growing body of research implicating microglia in the development of mood disorders. Beyond serving as the brain’s innate immune cells, microglia play a critical role in the healthy development and dynamic functioning of the brain, regulating processes from cell survival to synaptic stability [26–28]. Microglia-related impairments appear to underlie some of the pathological changes associated with psychiatric diseases, including depression [29, 30]. As a result, a neuroimmune hypothesis proposes that microglia dysfunction constitutes a potential risk factor or biomarker for these disorders [29–31]. This hypothesis is supported by data from various rodent stress models that report increased depressive-like behaviors concomitant with alterations in microglia number and/or activity throughout the brain, including hippocampus [29, 32, 33]. Moreover, the potent microglia inhibitor minocycline has been shown to mitigate many of the behavioral deficits in these models, as well as some of the underlying microglia alterations [34–49].

Taken together, these studies suggest that microglia play a role in the pathophysiology of affective disorders, yet critical questions remain regarding how individual differences in microglia relate to underlying temperament. Given that our transcriptional profiling data indicated elevated microglial gene expression in naïve bLRs [25], we hypothesized that bLR phenotype may be due, at least in part, to a steady-state of microglia over-activation. To address this question, we administered minocycline and characterized its impact on the phenotypic behaviors of our bred lines. We then completed a detailed anatomical analysis of hippocampal microglia populations in bHRs and bLRs and found unique morphological profiles associated with the divergent temperaments.

## Materials and methods

### Animals

Subjects were male Sprague Dawley rats acquired from our in-house breeding colony at the Michigan Neuroscience Institute (generations 52–65). The initiation and maintenance of our breeding lines are described in detail elsewhere [9] and in Supplementary Materials. Briefly, adult rats were screened for locomotor response to a novel environment, and the top and bottom 20% of responders were used to breed bHR and bLR lines, respectively. These lines have been maintained over several generations, resulting in highly stable and predictable divergent phenotypes. All experimental procedures were approved by the University Committee on the Use and Care of Animals at the University of Michigan and were conducted in accordance with the National Institute of Health Guide for the Care and Use of Laboratory Animals (2011).

### Quantitative reverse transcription-PCR (qPCR)

To determine baseline differences in gene expression, whole hippocampus was collected from adult bHRs and bLRs (60–75 days old; *n* = 10/line) under non-stress conditions. RNA was extracted using the RNeasy Mini Kit (Qiagen, Hilden, Germany), and cDNA was synthesized using iScript cDNA Synthesis Kit (Biorad, Hercules, CA). Amplification reactions were performed using a BioRad Cycler with SYBR Green detection. Samples were run in duplicate, and group differences in mean quantification cycle (Cq) values for each target gene were calculated using the ΔΔCq method [50], with glyceraldehyde-3-phosphate dehydrogenase (*GAPDH*) as the reference gene.

### Minocycline administration

Systemic minocycline treatment was used to broadly inhibit microglia. Minocycline hydrochloride (Sigma Aldrich, St. Louis, MO) was dissolved in drinking water (1 mg/ml, pH adjusted to ~7.4) to achieve an estimated daily dose of 60–100 mg/kg. For all behavioral experiments, minocycline (or tap water for controls) began when rats were 60–67 days old, was administered 14 days prior to the initiation of behavioral testing (15^th^ day), and continued throughout testing. The treatment regimen was chosen based on previous studies showing behavioral effects using similar doses and timing [39, 40]. Minocycline/water consumption and body weight were tracked over the course of the experiment. To confirm dosing, circulating plasma levels of minocycline were measured in a subset of subjects at the end of the experiment using a commercially available tetracycline enzyme immunoassay (Perkin Elmer, Akron, OH) following kit instructions [39].

### Behavioral testing

Initial studies were performed to test the effects of minocycline on one anxiety measure (elevated plus maze, EPM) and one depressive-like measure (forced swim, FS) in both bHRs and bLRs (*n* = 8/treatment group/line). Additional minocycline studies extended the characterization of minocycline effects in the highly sensitive bLRs (*n* = 12/treatment group), using multiple behavioral assays: novelty suppressed feeding (NSF), open field (OF), social interaction (SI), sucrose preference (SP), and FS. All behavioral tests were conducted between 9:00 AM–1:00 PM, and sequential tests were separated by 24–72 h. Behavioral coding was done by a researcher blinded to the identity of the subject. See Supplementary Materials for detailed descriptions of testing procedures.

### Iba1 immunohistochemistry

To determine baseline patterns of microglia, a separate group of experimentally naïve adults (65–75 days old; *n* = 8/line) were transcardially perfused for brain tissue fixation with minimal disturbance. Coronal brain sections (40‐μm thickness) were collected using a cryostat (−20 °C, 1:6 series) and stored in cryoprotectant-antifreeze solution. A single, randomly selected series of sections from each subject were processed using standard, free-floating immunohistochemical procedures [51]. Microglia were labeled using a primary antibody raised against the ionized calcium binding adapter molecule 1 (Iba1, FUJIFILM Wako Chemicals, Richmond, VA, concentration 1:70,000) and visualized with nickel-enhanced diaminobenzidine reaction. Iba1 is a microglia-specific marker that stains the cell body as well as the full extension of cell processes [52], allowing analysis of key morphological features known to reflect functional status [53].

For microscope analyses, live images were analyzed with a Leica DMR microscope attached to a digital camera and a three-axis motorized stage. The researcher performing the analysis was blinded to subject identity/group.

#### Iba1 staining density

We first calculated optical density of Iba1 immunostaining in bHRs and bLRs. Slides were scanned (PathScan Enabler; Meyer Instruments) and converted into 16-bit grayscale files. ImageJ software (NIH) was used to quantify the optical density of Iba1 staining. Dorsal hippocampus was outlined in four consecutive sections per subject (Atlas levels 28–34) [54]. Thresholding was applied (*Moments function*), and the number of pixels surpassing threshold was calculated as a percentage of the region outlined. This method has been used to capture microglia soma and processes and reflect density of Iba1 signal [55]. Density values were averaged across all sections to generate a single value/subject.

#### Microglia number

To compare the total numbers of microglia, non-biased stereological quantification was performed using the Optical Fractionator probe within Stereo Investigator software (MicroBrightField Bioscience, Williston, VT). For each subject (*n* = 7/line), four hippocampal sections were analyzed unilaterally (counterbalanced for comparable sampling of left (*n* = 3) and right (*n* = 4) hemispheres). The Cornu Ammonis 1 and 3 (CA1, CA3) and dentate gyrus (DG) sub-regions were outlined according to anatomical landmarks using a 2.5X objective and were analyzed separately. Cells were counted using a 40X dry-objective lens. Stereological sampling parameters (Table S1) were determined through pilot studies, ensuring sufficient sampling and Gundersen–Jensen error coefficients below 0.10. Iba1-positive cells were identified as having dark, blue–black somatic staining, and were excluded from counting if their cell body contacted the left or bottom edges of the counting frame [56]. A categorical assessment of microglia state [53] was assigned to each cell: *Ramified*: small cell body, with long/thin processes; *Reactive*: larger cell body, with few, stout processes; and *Amoeboid*: enlarged, round cell body, with minimal/no processes.

#### Microglia area

The size of microglia, both soma and processes, is dynamic, and shifts in cell area can provide insight into the functional state of the cell [53]. To generate quantitative estimates of microglia size in a population-wide (hundreds of cells/subject) and non-biased manner, we applied the Nucleator probe (isotropic, eight rays) during stereological sampling procedure [55, 57]. As each cell was selected for counting, a ray of eight lines was applied from the cell’s center. Points of intersection along the rays were used to generate two area measurements: (1) intersections with the edge of the cell body were used to estimate *soma area* and (2) intersections with the most distal extent of the cell processes were used to estimate *territory area*.

#### Microglia morphology

To further explore the morphological characteristics of microglia, we generated full reconstructions of a subset of ramified microglia from bHRs and bLRs using Neurolucida software (MicroBrightField Bioscience). Cells were selected from systematically placed circular regions of interest (ROI) within CA1, CA3, and DG on a single section per subject (*n* = 8/line). Five cells were selected per ROI (total: 40 cells /ROI/line). Individual ramified cells were selected based on a visible full cell body that was distinguishable from nearby cells, with no major disconnections of processes. Using a 63x-oil objective (1.32 numerical aperture), the edge of the cell body was first traced at a single plane of focus. Each process was then traced, with both the width of the process and plane of focus adjusted incrementally, until all visible processes were reconstructed. Reconstructions were analyzed using Neurolucida Explorer Software (MicroBrightField Bioscience). During data processing, 4 cells (two from bHRs; two from bLRs) were identified as extreme outliers in multiple measures (Grubb’s test, *p* < 0.05) and therefore removed from the analyses (final *n* = 38 cells/line).

### Statistical analysis

All data were analyzed with SPSS v26 (IBM, Armonk, NY). Equality of variance was assessed using either Levene’s or Mauchly’s test. For qPCR data, −ΔΔCq values were analyzed using Welch’s independent *t*-tests due to unequal variance. For the bHR/bLR minocycline study, 2-way ANOVAs were performed with Line*Treatment as between-subjects factors. Significant interactions were explained using simple-effects analyses. For the bLR-only minocycline study, independent *t*-tests (two-tailed) compared treatment groups. For Iba1 analyses, data were first analyzed with mixed-factors ANOVAs to determine if the effects of line varied across sub-region analyzed (CA1, CA3, DG). In the presence of a significant interaction, results were analyzed and presented separately for each sub-region. Line differences in Iba1 measures were determined using independent samples *t*-tests (two-tailed), with Bonferroni correction where appropriate. Full statistical values are presented in Supplemental Materials (Tables S2–S8) and in Figure Legends.

## Results

### Hippocampal expression of microglia-signaling genes was altered by selective breeding for novelty responses

**Fig. 1:**
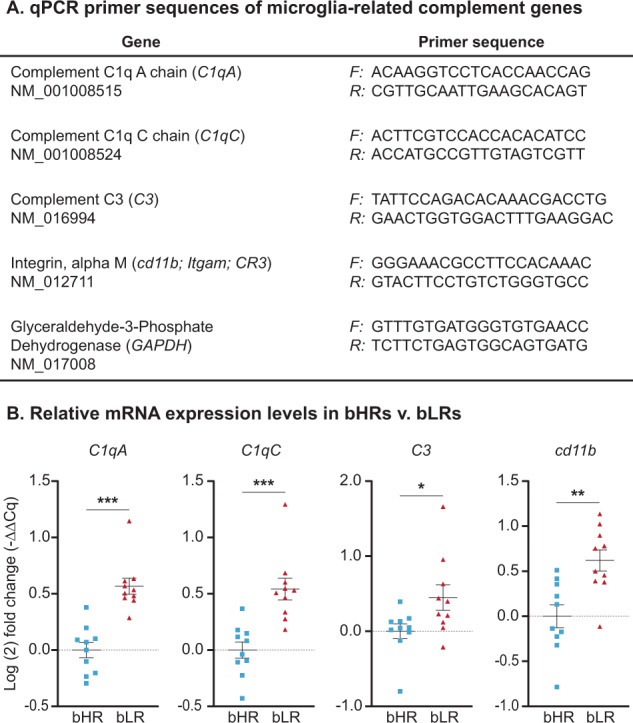
Differential patterns of microglia-related gene expression in hippocampus following selective breeding. **A** Primer sequences of genes used for qPCR experiments. All primers were validated in-house, and expression levels of *GAPDH* (reference gene) were not significantly different between bHRs and bLRs (Supplemental Materials, Fig. S1). **B** Elevated hippocampal expression (Log(2) fold change) for several genes within the classical complement cascade was found in bLRs compared to bHRs. Data are expressed as mean ± SEM, with scatter plots overlaid to show individual data points. Welch’s *t*-tests: bHR v. bLR, ***C1qA: *t*_17.92_ = 33.713, *p* < 0.001; ***C1qC: *t*_16.63_ = 20.305, *p* < 0.001; *C3: *t*_14.52_ = 5.258, *p* = 0.037; **Cd11b: *t*_17.89_ = 12.874, *p* = 0.0012.

Our first goal was to confirm and extend findings from our recent bHR/bLR meta-analysis showing differential expression of microglial signaling genes in hippocampus [25]. qPCR measured expression levels of multiple genes within the classical complement cascade, including cascade components *C1q* and *C3*, and the receptor molecule *cd11b* (Fig. 1A). In the brain, this pathway represents a major signaling mechanism for microglia, reflecting inflammatory and macrophagic responses, as well as broad functions related to neurodevelopment and plasticity [58, 59]. Similar to our meta-analysis, we found that bLRs had higher hippocampal expression than bHRs of multiple genes within the complement pathway (Fig. 1B), including elevated expression of the initiator molecules *C1qA* (*p* < 0.0001) and *C1qC* (*p* = 0.0003) [25]. We extended this pattern to include the downstream effector *C3* (*p* = 0.0372) and complement receptor molecule *cd11b* (*p* = 0.0021). These data implicate microglia as a source of variation that may underlie divergent behavioral temperaments.

### Minocycline administration improved measures of emotional and social behaviors in animals with an inhibited temperament

**Fig. 2:**
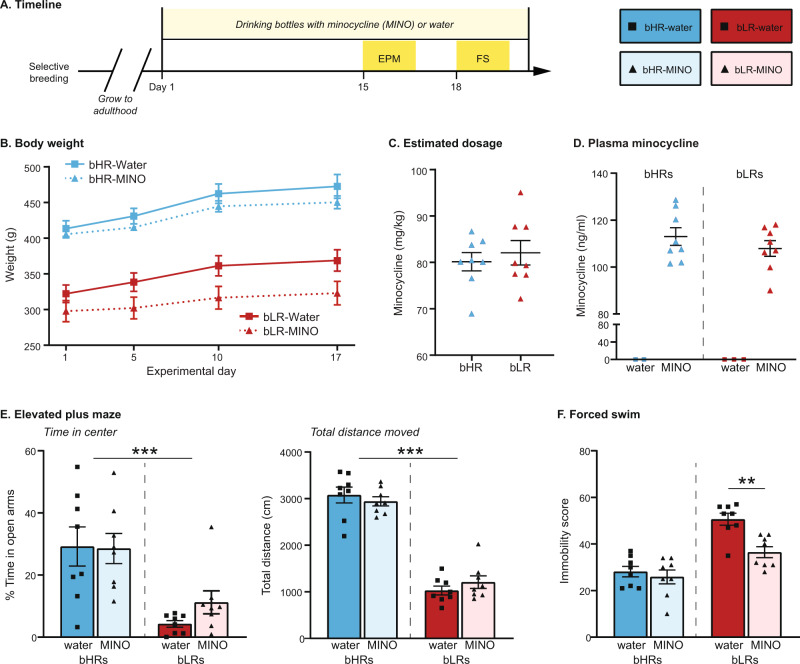
Comparison of minocycline effects in bHRs and bLRs. Minocycline effects were initially assessed for a single proxy measure of anxiety (Elevated Plus Maze, EPM) and depression (Forced Swim, FS) in both bHRs and bLRs. **A** Schematic of administration and testing timeline; Day 1 = first day of minocycline administration. **B** Body weight was measured periodically throughout minocycline administration and revealed faster growth rates in bHRs (*F*_1,28_ = 74.642, *p* < 0.001). Minocycline administration reduced weight gain overall (*F*_1,28_ = 4.617, *p* = 0.040), with no significant Line*Treatment interaction. **C** Minocycline dosages (mg/kg) were estimated by dividing body weight by consumption volumes and were similar in bHRs and bLRs. **D** Plasma minocycline measurements also indicated similar levels of circulating minocycline between bHRs and bLRs at the end of the experiment. **E** In the EPM, bLRs spent less time in the open arms overall (***F_1,28_ = 17.423, *p* < 0.001), but there was no effect of minocycline in either line. There was also an overall line difference in total distance moved (***F_1,28_ = 216.822, *p* < 0.001), but again, no effect of minocycline in either line. **F** In the forced swim test, a Line*Treatment interaction (*F*_1,28_ = 5.418, *p* = 0.027) showed that minocycline reduced immobility scores in bLRs (**t_14_ = 4.048, *p* = 0.001) but not in bHRs (*t*_14_ = 0.605, *p* = 0.555). Data are expressed as mean ± SEM, with scatter plots overlaid to show individual data points.

To test whether microglial activation plays a role in our selective phenotypes, we administered minocycline in the drinking water [39, 40]. Minocycline is a tetracycline antibiotic that readily crosses the blood-brain barrier and inhibits microglial activation in the brain following peripheral administration [39, 40, 49, 60–62]. We first compared the effects of minocycline on select measures of anxiety (EPM) and depressive-like (FS) behavior in bHRs and bLRs (Fig. 2A). Body weight (Fig. 2B) was higher overall in bHRs compared to bLRs (*p* < 0.001), and minocycline administration was associated with reduced weight (*p* = 0.040), although there was no significant Line*Treatment interaction (*p* > 0.05). Importantly, fluid intake measurements indicated comparable estimated dose ranges within 68–98 mg/kg (Fig. 2C), and plasma minocycline levels confirmed equivalent levels of circulating minocycline between the lines (Fig. 2D).

Consistent with their previously described phenotypes [15, 20], bLRs displayed high anxiety in the EPM (Fig. 2E), spending less time in the open arms (*p* < 0.001) and moving less overall (*p* < 0.001) than compared to bHRs. Minocycline had no effect on EPM measures in either line (all *p* > 0.05). For FS (Fig. 2F), bLRs again showed phenotypically high immobility scores compared to bHRs (*p* < 0.001), but there was a significant Line*Treatment interaction (*p* = 0.027): minocycline reduced immobility in bLRs (*p* = 0.001) but had no effect in bHRs (*p* = 0.555). These results suggest that minocycline has an anti-depressant effect in rats that display high levels of depressive-like behaviors at baseline.

**Fig. 3:**
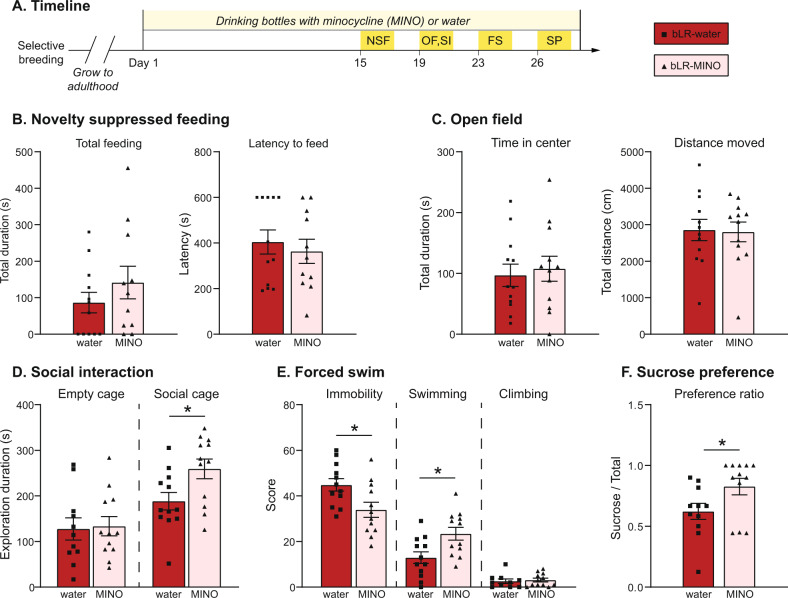
Minocycline shifts multiple aspects of bLR phenotype. A separate cohort of bLRs was given an extended testing sequence following minocycline administration. **A** Schematic of administration and testing timeline, Day 1 = first day of minocycline. Minocycline did not affect bLR anxiety measured by novelty suppressed feeding (**B**) or open field (**C**) (all *p* > 0.05). In the social preference test (**D**), minocycline-treated bLRs spent more time exploring the cage containing the social stimulus compared to control bLRs (*t_22_ = 2.454, *p* = 0.023). **E** In the Forced Swim challenge, minocycline decreased immobility scores (*t_22_ = 2.754, *p* = 0.012), specially through an increase in swimming behavior (*t_22_ = 2.775, *p* = 0.011). **F** Minocycline also increased sucrose preference (*t_22_ = 2.155, *p* = 0.042). Data are expressed as mean ± SEM, with scatter plots overlaid to show individual data points. Abbreviations: FS forced swim; NSF novelty suppressed feeding; OF open field; SI social interaction; SP sucrose preference.

Additional studies were performed to confirm the anti-depressant effect of minocycline in bLRs, as well as to expand the testing sequence to include a broader array of emotional measures. For these studies (Fig. 3A), body weight and fluid intake measured throughout the experiment indicated systemic doses of 66–84 mg/kg (mean = 75 mg/kg; Fig. S2). Consistent with results from the EPM, minocycline did not alter bLR anxiety-like behavior measured in NSF or OF (Fig. 3B, C, *p* > 0.05). However, minocycline significantly increased social exploration (Fig. 3D; *p* = 0.023), an effect that was not observed with the empty stimulus cage (*p* = 0.726), indicating specificity for social motivation. For FS, control bLRs again displayed high immobility scores (Fig. 3E), and minocycline again reduced this measure (*p* = 0.012), increasing swimming behaviors specifically (*p* = 0.011). Finally, minocycline increased the preference to consume sucrose solution over water (Fig. 3F*, p* = 0.042), without altering the total volume of liquid consumed (not shown, *p* = 0.621). In summary, inhibition of microglia with minocycline treatment shifted social and depressive-like behaviors in bLRs.

### Divergent temperaments were associated with shifts in the territory area of hippocampal microglia, despite similar total numbers of microglia

**Fig. 4:**
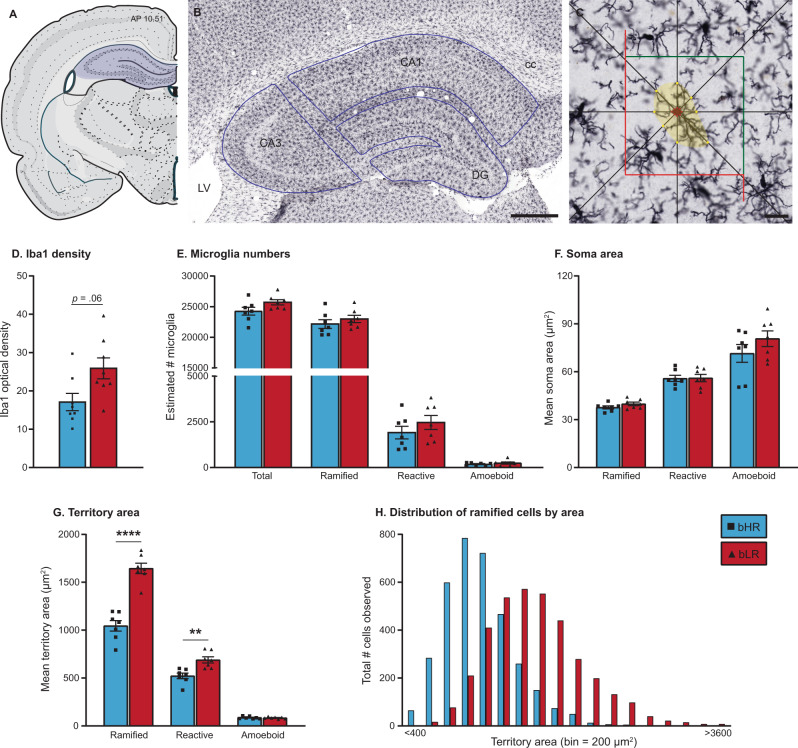
Comparison of microglia density, numbers, and area in hippocampus of bHRs and bLRs. **A** Coronal sections of dorsal hippocampus were matched for rostrocaudal level using a Rat Brain Atlas [54] (Atlas levels 28–34). **B** Photomicrograph showing immunohistochemical staining of microglia marker Iba1. The different sub-regions were sampled separately during stereological sampling and were drawn using anatomical landmarks (blue lines). Scale bar = 500 µm. **C** Recreated depiction of workflow using Optical fractionator and Nucleator probes. Cells were counted if soma was within the sampling grid (red and green lines). When a cell was selected, the Nucleator probe applied eight rays from the center of the cell, and points of intersection were used to estimate soma area (red markers) and cell territory (yellow markers, distal extensions of cell processes). Scale bar = 20 µm. **D** Optical density measurements revealed a trend for higher density of Iba1 staining in hippocampus of bLRs compared to bHRs. **E** Stereological estimates of total microglia numbers were similar between bHRs and bLRs, regardless of cell type. **F**, **G** Soma and territory areas generated using Nucleator Probe were averaged for each subject, according to morphological category (*Ramified, Reactive, Amoeboid*). Although soma area measurements (**F**) were not different between the lines, estimates for territory area (**G**) were larger in bLRs compared to bHRs, specifically in ramified (***t_12_ = 7.709, *p* < 0.001) and reactive microglia (**t_12_ = 3.843, *p* = 0.002). **H** A frequency distribution of territory area for all ramified microglia illustrates a normal distribution in both lines, but a notable right-ward shift in territory size of ramified microglia from bLRs compared to bHRs. Data in **D**–**G** are expressed as mean ± SEM, with scatter plots overlaid to show individual data points. Abbreviations: CA1 and CA3 = Cornu Ammonis 1 and 3; cc corpus callosum, DG dentate gyrus, LV lateral ventricles. Data are expressed as mean ± SEM, with scatter plots overlaid to show individual data points.

We predicted that the extreme differences in inborn temperament in bHR/bLR rats were associated with underlying variation in the baseline status of microglia, either in the total number of microglia, or in their activation state. To test this prediction, we generated a detailed, anatomical comparison of hippocampal microglia populations in a separate cohort of experimentally naïve bHRs and bLRs using immunohistochemical labeling of microglia (Fig. 4A–C). We focused our anatomical analysis on the hippocampus, as this region is characterized by robust bHR/bLR differences in gene expression, particularly in genes related to microglia function [25]. For this cohort, body weights measured at the time of perfusion were not significantly different between bHRs and bLRs (Fig. S3). When comparing Iba1 staining density, we detected a trend for increased staining in bLRs compared to bHRs (Fig. 4D; *p* = 0.056). We then used non-biased stereological sampling techniques to estimate the total microglia number within each sub-region. The effects of line did not vary by sub-region (all Line*Region: *p* > 0.05); data were therefore combined across sub-regions for each subject. The estimates for total number of microglia were similar between bHRs and bLRs, with a trend for higher total microglia in bLRs (Fig. 4E; *p* = 0.085). When estimates were broken down by morphological category, for both lines, most microglia were classified as ramified (mean ± SEM: bHRs = 91.34 ± 1.44%; bLRs = 89.43 ± 1.63%). Again, estimated total numbers for each category were not significantly different between bHRs and bLRs (Fig. 4E; *p* > 0.05). Importantly, these results indicate that the observed differences in microglial gene expression were unlikely to reflect differences in total microglia numbers or shifts in the bLR microglia population toward the reactive/amoeboid states.

Given the relationship between microglia size and function [53], soma and territory area measurements were generated for every microglial cell counted within the stereological sampling procedure, generating quantitative size estimates for a range 480–624 total cells/subject (mean cells/subject ± SEM: bHRs = 536 ± 14; bLRs = 567 ± 11; Table S6). The effects of line did not vary according to sub-region (Table S7); data were therefore collapsed across sub-regions. In general, area estimates were consistent with criteria used for morphological categories, with increasing soma area and decreasing territory area from ramified, to reactive, to amoeboid cells (Fig. 4F, G). Although there were no significant differences between bHRs and bLRs in soma area for any microglia category (Fig. 4F; all *p* > 0.05), territory areas were larger in bLRs compared to bHRs for both ramified (*p* < 0.0001) and reactive (*p* = 0.0023) microglia (Fig. 4G). To further illustrate differences in the size of ramified microglia, we generated frequency distributions of the total number of ramified microglia across the range of territory areas (binned by 200 µm [2] increments; Fig. 4H). These plots demonstrated a right-ward shift in the distribution of territory area of hippocampal microglia in bLRs: the bLR microglia population had larger mode and larger maximum measurements compared to the bHR population.

### Detailed morphological analyses indicated hyper-ramification of microglia in bLRs compared to bHRs

**Fig. 5:**
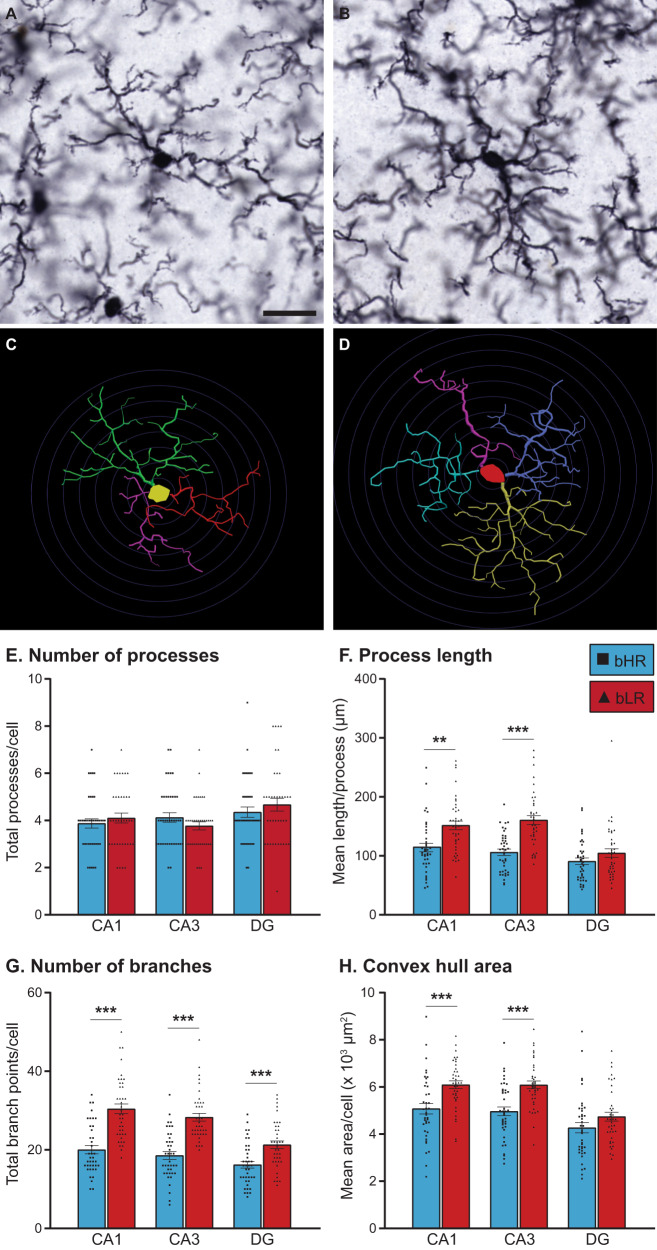
Detailed morphological analyses of ramified microglia in bHRs and bLRs. High-magnification photomicrographs (63x, oil) of typical ramified microglia from bHR (**A**) and bLR (**B**), with Neurolucida reconstructions for each cell shown in (**C**) and (**D**), respectively. Scale bar = 20 µm; Concentric circles applied at 5 µm increments in **C** and **D** for length comparisons. Reconstructions were analyzed using Neurolucida Explorer Software (MicroBrightField Bioscience). The main measurements are summarized here, with a full listing provided in Supplemental Materials. Although microglia from each line had similar totals of primary branches (**E**), bLR microglia had longer branches in CA1 (**t_74_ = 3.322, *p* = 0.001) and CA3 (***t_74_ = 5.765, *p* < 0.001) (**F**), and more branch points across all regions (***CA1: *t*_74_ = 6.267, *p* < 0.001; ***CA3: *t*_74_ = 7.012, *p* < 0.001; ***DG: *t*_74_ = 4.022, *p* < 0.001) (**G**), compared to microglia from bHRs. **H** Convex hull calculations, which reflect the overall territory area of the cell, were larger in bLRs in CA1 (***t_74_ = 3.909, *p* < 0.001) and CA3 (***t_74_ = 4.869, *p* < 0.001). Data are expressed as mean ± SEM, with scatter plots overlaid to show individual data points.

Intriguingly, the larger territory area measurements of ramified cells in bLRs are consistent with elongation of microglia processes that occurs when cells undergo hyper-ramification. This morphology has been observed when animals are exposed to certain forms of stress or environmental challenges and is thought to reflect an intermediate stage of the microglia response [39, 40, 63]. To directly assess the possibility of hyper-ramification in bLRs, we generated detailed reconstructions for a subset of ramified microglia from bHRs and bLRs. For this data set, line effects were not homogenous across sub-region (Table S8); data were therefore analyzed separately for CA1, CA3, and DG. Figure 5A, B show high-magnification photomicrographs of typical ramified cells from each line, along with their associated reconstructions generated using Neurolucida (Fig. 5C, D). Overall, the morphological analyses were consistent with the process area estimates and provide more detailed information regarding microglia complexity. Although the total number of primary processes/cell was similar between lines (Fig. 5E; all *p* > 0.05), these processes were longer in bLRs compared to bHRs (Fig. 5F; CA1, *p* = 0.001; CA3, *p* < 0.001), and there were higher numbers of branch points/cell in bLRs (Fig. 5G; all sub-regions *p* < 0.001). As a result, the area of the territory encompassed by the entire cell (estimated using convex hull analysis) was larger in bLRs compared bHRs (Fig. 4H), specifically within CA1 (*p* < 0.001) and CA3 (*p* < 0.001). Soma area from these reconstructions also revealed a significantly larger soma area in bLRs within CA1 and CA3 (Table S8). Sholl analyses revealed consistent increases in process complexity across the microglia, but particularly within proximal portions of the cell (Fig. S4). In summary, microglia from bLRs had longer processes, with more complicated branching patterns, and larger overall territories, an overall morphological pattern consistent with hyper-ramification.

## Discussion

We used a selective breeding model to examine the role microglia play in contrasting temperaments. The bHR and bLR lines reflect a range of divergent emotional and coping responses, including anxiety- and depression-like behaviors [3]. After identifying elevated expression levels of several microglia-related genes in bLR hippocampus, we hypothesized that microglia play a role in shaping their uniquely inhibited phenotype. We found that the microglia blocker minocycline improved several emotional measures in bLRs, consistent with a general functional role for microglia in key aspects of their phenotype. To determine whether underlying baseline differences in microglia cells were associated with selective breeding, we performed detailed anatomical comparisons of microglia in a separate cohort of naïve adults. These analyses found that, although bHRs and bLRs had similar total numbers of microglia within the hippocampus, cells from bLRs were characterized by a hyper-ramified morphology, reflective of an intermediate stage of microglia activation. This study provides evidence linking variation in inborn temperament with differences in morphological status of microglia and highlights the importance of considering microglia as key players in the enduring aspects of emotional responsivity.

### Animals with divergent temperaments express different levels of microglia-signaling genes within the hippocampus

Over seventeen years of selective breeding, multiple molecular pathways have been identified as underlying bHR/bLR temperaments [3]. Collectively, these studies confirm a role for the hippocampus in bHR/bLR phenotypes, outlining key differences in the structure and function of a region known to regulate emotional processing [3, 14, 15, 20, 24, 64]. A recent meta-analysis of eight transcriptional datasets, spanning 43 generations, implicates a broad range of hippocampal transcripts underlying bHR/bLR divergence [25]. Of these potential candidates, microglia-related genes were found to be highly upregulated in bLRs compared to bHRs, a pattern that complements emerging theories of microglia mechanisms of mood disorders [29]. One of the top genes identified, *C1qA*, plays a critical role in microglia signaling as the initiator of the classical complement cascade [58, 65]. The meta-analysis also showed that a genetic variant near *C1qA* segregates bHR and bLR lines, providing evidence for genetic determinants of the differential expression profiles [25].

The current qPCR analyses confirmed the differential expression of *C1qA* and *C1qC* observed previously and extended these results to demonstrate that additional molecules within the classical complement pathway are also upregulated in bLRs, including the *C3* ligand and a component of its receptor *cd11b* (CR3). In contrast, we have not previously observed increased expression of traditional neuroinflammatory markers, including pro-inflammatory cytokines [25]. This finding is consistent with reports that bHR/bLR lines have mostly similar levels of plasma cytokines [66] and suggest that bHR/bLR differences in microglia gene expression may reflect non-pathological, nonimmune functions. Microglia signaling plays a role in many aspects of healthy brain function, and the complement cascade pathway has been linked to synaptic pruning in development and plasticity throughout life [58, 65, 67]. Determining the specific role for the complement pathway in regulating emotional phenotypes in our selective breeding model is a compelling avenue for future studies.

### Minocycline treatment alters key aspects of the inhibited phenotype of bLRs

Research into the use of minocycline for treatment of affective disorders is expanding, and a meta-analysis of random controlled clinical trials of minocycline suggest good therapeutic potential in patients with major or bipolar types of depression [68, 69]. The clinical trials are supported by many rodent studies that report anti-depressant effects of minocycline [34–49]. Although some anti-depressant effects have been observed in naïve animals [35], most studies report behavioral changes specifically following a pro-depressant challenge (e.g., chronic stress, immune stimulation). The fact that minocycline typically has no effects in control animals suggests that outbred strains may not be sensitive to the effects of minocycline at baseline conditions. However, the natural variation that exists within outbred strains leaves the possibility that minocycline may be efficacious in certain sub-populations, but those effects are not detected when averaged across the whole group.

In support of this interpretation, we found that minocycline had good efficacy in bLRs, even in the absence of any overt stress or challenge. Similar to the results described here, Schmidtner and colleagues also showed that minocycline reduces depressive-like behaviors in rats bred for high anxiety behavior (HAB), but not in the non-anxious line [70]. Also consistent with the current results, minocycline altered HAB behavior in the forced swim and social exploration tests, with no changes in anxiety measures. The current study expanded on these findings to show that minocycline can also increase sucrose preference in vulnerable animals, implying reduced anhedonia. Together, the results from these selective breeding models highlight the importance for understanding how inborn traits may shape the expression of emotional behaviors and sensitivity to therapeutics.

Minocycline has well-established functions of suppressing microglia proliferation and signaling [60–62]. The tight link between microglia and depressive behavior observed in various animal models [36], and the consistent shift in both behavior and microglia following minocycline administration [39, 46, 49], suggest a functional relationship. It is important to note, however, that minocycline may have broad effects, with direct and indirect consequences extending beyond microglia function. In addition to its anti-inflammatory effects, potential anti-depressant mechanisms of minocycline include regulation of neurogenesis, antioxidation, apoptosis, and excitatory toxicity processes [36, 71]. Minocycline may even be acting outside of the brain by changing the composition of the gut microbiome [70], although a recent study suggests that bHR/bLR temperaments are not associated with baseline differences in microbiota [66]. The precise mechanism through which minocycline alters emotional behavior will need to be explored further in this model and may involve complex interactions among multiple pathways.

### Morphological status of microglia is shifted in animals with divergent temperaments

Microglia are dynamic players in the brain, and their function can be reflected by alterations in total numbers, as well as functional state. When at rest, microglia maintain a ramified structure, with long motile processes actively surveying their local territory [53, 72, 73]. Upon stimulation, microglia morphology shifts along a continuum, progressing through a reactive phase, with shortening and thickening of processes, to the fully activated amoeboid (macrophagic) phase, with enlarged soma and full retraction of processes [63, 74, 75]. Descriptions of microglia “activation” typically refer to these reactive/amoeboid morphologies, although the field has begun to recognize additional morphologies within the spectrum of microglia responses [63]. In particular, “hyper-ramification” is characterized by an elongation of processes and increase in branch complexity and is thought to reflect an early stage of activation, or perhaps responses to relatively less intense (non-pathological) stimulation [39, 40, 63]. A major goal of the current study was to compare bHR/bLR microglia numbers and morphological characteristics, to determine how selective breeding determines the tone of microglia populations.

Interestingly, our anatomical studies revealed that total microglia numbers were mostly unaffected by selective breeding. bHRs and bLRs also had similar numbers of cells in reactive or amoeboid states. These results suggest that the elevated expression of microglia-related genes observed in bLR hippocampus does not reflect underlying differences in total microglia number or shifts toward fully activated states. Instead, we found that ramified microglia from bLRs had longer processes, with more complicated branch patterns, than microglia from bHRs. bLR microglia also had larger territories, a difference detected across large numbers of cells measured during stereological sampling and in the more detailed reconstructions of a subset of cells. This specific morphological pattern is consistent the hyper-ramified stage of activation [39, 40, 63]. In line with this interpretation, cd11b, which is elevated in bLR hippocampus (Fig. 1) has been linked not only to microglia activation generally [53], but also to hyper-ramification states specifically [76]. Thus, at both the gene expression and morphological levels, our data indicate shifted reactivity of bLR microglia within hippocampus.

Several studies report evidence of hyper-ramified microglia following environmental challenges. Manipulations that provoke hyper-ramification include various forms of chronic stress, including restraint, inescapable swim, foot shock, and prenatal dexamethasone exposure [39, 40, 63, 77–81]. Wild-type mice exposed to repeated forced swim display increased despair behavior, concomitant with increased hyper-ramification within dentate gyrus, and both the behavioral and microglia effects are abrogated by treatment with the anti-depressant venlafaxine [78]. The present findings provide novel evidence associating *baseline* states of hyper-ramification to emotional vulnerability. Indeed, in our anatomical studies, there was no overt experimental stress or manipulation, and we did not see evidence for later stages of microglia activation. Moreover, the microglia profile associated with bLR’s bred temperament closely matches the microglia profiles described following stress, a compelling convergence given the known role of stress in mood disorders [82]. Although ramification patterns following minocycline treatment were not assessed in the current study, previous studies demonstrate that minocycline reduces stress-induced hyper-ramification of prefrontal microglia [39, 40], suggesting a potentially similar mechanism in bLRs.

What are the consequences of hyper-ramification on microglia physiology and function? Hyper-ramification has not been associated with markers of neuroinflammation or neurotoxicity [39, 40, 83], suggesting a link with non-diseased conditions. This morphology may reflect a transitional step intermediate to full activation phases, or it may represent its own distinct endpoint. One possibility is that hyper-ramified microglia are in a “primed” state and would have heightened responses to future perturbations. In a mouse model of post-traumatic stress, increased hyper-ramification is associated with a decrease in spine density [80], consistent with a hypothesis of over-active macrophagic pruning by hyper-ramified microglia. In fact, hyper-ramification has been associated with increased expression of C1q [80] and CR3 [74, 83, 84], which serve synaptic pruning functions via their roles in the classical complement cascade [65, 67, 85, 86]. Alternatively, it is possible that hyper-ramified microglia are arrested in this phase, and subsequent physiological responses may be impaired. In support of this possibility is the fact that microglia can develop extreme hyper-ramification in the aged brain, resulting in entanglement and dysfunction [87]. Although we did not observe evidence for microglia tangles, it is interesting to consider the possibility that inborn differences in microglia morphology may reflect an accelerated aging process.

### Conclusions and further considerations

Individual differences in temperament represent stable traits that broadly shape behavior and reactivity in an ongoing manner. Understanding the genetic and neurobiological mechanisms that underlie temperamental differences is essential to uncovering the biology of mood and other affective disorders. Through our selective breeding model, we have characterized divergent phenotypes that are hereditable, highly stable, and track with behavioral vulnerability across multiple measures. This model has allowed us to explore the multiple, polygenic factors that regulate emotional temperament, and the current data suggest that underlying variation in microglia tone should be added to the list of potential contributing factors.

While these studies provide novel evidence linking an inhibited phenotype to a unique microglia profile, the functional significance of hyper-ramification per se on bLR behavior requires further investigation. Hyper-ramification may function causally to regulate behavior, or this unique morphology may represent a novel biomarker for emotional vulnerability. Critical questions remain regarding how the hyper-ramified status in bLRs develops, and in turn, what that means for subsequent responses to environmental challenges known to impact behavior. It will be important to use a range of tools to mechanistically define the role of a chronic hyper-ramified state in temperament.

## Supplementary information

Supplemental Materials
